# The contribution of wildland fire emissions to deposition in the U S: implications for tree growth and survival in the Northwest

**DOI:** 10.1088/1748-9326/abd26e

**Published:** 2021-01-29

**Authors:** Shannon N Koplitz, Christopher G Nolte, Robert D Sabo, Christopher M Clark, Kevin J Horn, R Quinn Thomas, Tamara A Newcomer-Johnson

**Affiliations:** 1 Center for Environmental Measurement and Modeling, US EPA, Research Triangle Park, NC, United States of America; 2 Center for Public Health and Environmental Assessment, US EPA, Washington, DC, United States of America; 3 Department of Forest Resources and Environmental Conservation, Virginia Tech, Blacksburg, VA, United States of America; 4 Center for Environmental Measurement and Modeling, US EPA, Cincinnati, OH, United States of America; 5 Current address: Office of Air Quality Planning and Standards, US EPA, Research Triangle Park, NC, United States of America

**Keywords:** wildland fires, N deposition, ecosystem impacts

## Abstract

Ecosystems require access to key nutrients like nitrogen (N) and sulfur (S) to sustain growth and healthy function. However, excessive deposition can also damage ecosystems through nutrient imbalances, leading to changes in productivity and shifts in ecosystem structure. While wildland fires are a known source of atmospheric N and S, little has been done to examine the implications of wildland fire deposition for vulnerable ecosystems. We combine wildland fire emission estimates, atmospheric chemistry modeling, and forest inventory data to (a) quantify the contribution of wildland fire emissions to N and S deposition across the U S, and (b) assess the subsequent impacts on tree growth and survival rates in areas where impacts are likely meaningful based on the relative contribution of fire to total deposition. We estimate that wildland fires contributed 0.2 kg N ha^−1^ yr^−1^ and 0.04 kg S ha^−1^ yr^−1^ on average across the U S during 2008–2012, with maxima up to 1.4 kg N ha^−1^ yr^−1^ and 0.6 kg S ha^−1^ yr^−1^ in the Northwest representing over ~30% of total deposition in some areas. Based on these fluxes, exceedances of S critical loads as a result of wildland fires are minimal, but exceedances for N may affect the survival and growth rates of 16 tree species across 4.2 million hectares, with the most concentrated impacts occurring in Oregon, northern California, and Idaho. Understanding the broader environmental impacts of wildland fires in the U S will inform future decision making related to both fire management and ecosystem services conservation.

## 1. Introduction

While all ecosystems require nitrogen (N) to sustain healthy function and productivity, too much N can be ecologically damaging (Bobbink et al 2010, Sutton et al 2011). Excessive N loading can also directly threaten human health, for example through the production of toxins in water bodies affected by harmful algal blooms (Anderson et al 2002). Atmospheric deposition contributes significantly to concentrations of N and other ecologically relevant compounds like sulfur (S) in both terrestrial and aquatic ecosystems, but the magnitudes of individual source contributions remain uncertain (Mahowald et al 2017). Wildland fires are a major source of atmospheric N and are increasing in severity in some areas—the 2020 wildfire season was one of the worst on record for much of the western U S. The goal of this work is to refine existing estimates of N deposition due to wildland fire emissions and to assess some of the ecological impacts in the U S.

Atmospheric sources of N and S include a variety of natural and anthropogenic processes (Behera et al 2013, Paulot et al 2013). Sulfur dioxide (SO_2_) and nitrogen oxide (NO_x_) emissions have been decreasing significantly in the US since the 1990s (USEPA 2018), driven largely by reduced emissions from the energy sector (De Gouw et al 2014). These decreasing anthropogenic emission trends have been reflected in observed decreases in total N and S deposition nationally (although deposition of reduced N has been increasing in some areas) (Li et al 2016).

Conversely, wildland fire activity has been increasing in parts of the U S since the 1980s (Dennison et al 2014), a trend that is expected to continue in some areas (Yue et al 2013, Barbero et al 2015, Holden et al 2018). Here, ‘wildland fire’ includes both wildfire and controlled burns performed for ecological land maintenance and wildfire suppression (i.e. prescribed fires). Fire suppression policies implemented in the early 20th century combined with increasing human expansion into the wildland-urban interface (WUI) have altered naturally occurring fuel distributions and other landscape features linked to fire risk across the U S (Moritz et al 2014, Calkin et al 2015). Recent assessments have identified the potential utility of deliberate burning in fire prone areas to reduce fuel loads and decrease the severity of future wildfire events (Vaillant et al 2009, Parks et al 2014), and the use of controlled burns has become a regular and important part of fire management practices in many areas (Kolden 2019).

Besides emitting primary carbonaceous aerosols and a wide range of volatile organic compounds, wildland fires also emit gaseous inorganic species such as NO_x_, ammonia (NH_3_), and SO_2_ (Akagi et al 2011, Benedict et al 2017). These gaseous precursors contribute to secondary ammonium sulfate and ammonium nitrate aerosol formation in the atmosphere, species which are eventually returned to the earth’s surface through depositional processes such as precipitation events. A portion of the initial gas phase NO_x_, NH_3_, and SO_2_ is also directly deposited to downwind ecosystems.

While much of the U S experiences some degree of air quality impacts from wildland fires (Kaulfus et al 2017), the most severe fire activity often occurs in the western U S (Barbero et al 2014). The western U S is also home to many sensitive, nutrient-limited ecosystems (Fenn et al 2003a). Though wildland fires have been a naturally occurring part of the western U S landscape dating back at least to pre-European settlement (Keane et al 2009, Falk et al 2011), decades of N deposition from fossil fuel combustion and industrial agriculture have pushed the nutrient status of many of these sensitive ecosystems up to or even above thresholds at which ecological damage occurs (Clark et al 2018). Consequently, even small changes in atmospheric deposition associated with wildland fire emissions today could have a heightened environmental impact, despite the longstanding role of fire in ecosystem functioning (figure S1 which is available online at stacks.iop.org/ERL/16/024028/mmedia).

Understanding the contribution of atmospheric sources to concentrations of N and other nutrients in vulnerable ecosystems is particularly important given the relatively rapid response of atmospheric deposition patterns to changing emissions (Sickles and Shadwick 2015, Li et al 2016). However, observational constraints of atmospheric N fluxes are limited, especially in the western U S (Fenn et al 2003b). To fill this gap, modeling tools have been used to estimate atmospheric N fluxes over the U S (Paulot et al 2013, Schwede and Lear 2014) and to assess which areas experience ecologically meaningful increases in N deposition from atmospheric sources (Ellis et al 2013, Lee et al 2016, Clark et al 2018). While these existing modeling studies are informative, many of these previous assessments were conducted with global fire emission inventories that likely underestimate wildland fire emissions in the U S (Koplitz et al 2018) and/or did not focus on individual source contributions.

An assessment of the contribution of wildland fires to N and S deposition is hampered by the lack of accurate and methodologically consistent long-term records of fire activity with sufficient temporal and spatial precision to estimate the associated emissions. In this study, we leverage wildland fire emission estimates and high-resolution regional air quality modeling conducted over a continuous 5-year period to provide more spatially detailed estimates of the contribution of wildland fire activity to nutrient deposition over the contiguous U S. While 5 years is inadequate to capture a comprehensive range of fire behaviors and meteorological conditions affecting smoke transport, this collection of fire-attributed model simulations represents, to our knowledge, (a) a novel high-resolution dataset for application across the contiguous U S, and (b) the best estimate of fire-related deposition over multiple years available. We then calculate how tree growth and survival rates are influenced by changes in N and S deposition from fires by applying species-level responses to N and S for 16 species of trees (Horn et al 2018) in the areas with the largest amounts of deposition from wildland fires.

## 2. Methods

### 2.1. CMAQ air quality modeling

Simulations with the Community Multiscale Air Quality (CMAQ) model version 5.0 (Appel et al 2013) were performed at 12 km horizontal resolution over the contiguous U S for the 2008–2012 period. CMAQ is a regional chemical transport model routinely used for a wide range of air quality and earth science applications (Ring et al 2018, Bytnerowicz et al 2019, Zhou et al 2019). Simulated deposition and species concentrations are evaluated frequently against available observations by the scientific community and with each model release version (Foley et al 2010, Appel et al 2011, 2013, 2017, Nolte et al 2015, Zhang et al 2018). CMAQ simulations were performed both with and without wildland fire emissions to isolate the contribution of wildland fire activity to atmospheric pollutant concentrations (Fann et al 2017, Rappold et al 2017, Wilkins et al 2018).

CMAQ was configured using the CB05TUCL chemical mechanism (Sarwar et al 2008, Whitten et al 2010) and the AERO6 aerosol module (Simon and Bhave 2012, Appel et al 2013, Nolte et al 2015). Wildland fire emission estimates were generated using the SmartFire-BlueSky framework. Fire activity information was principally derived from incident status summary (ICS-209) reports and Hazard Mapping System (HMS) fire detections (Schroeder et al 2008), with additional data sources available for certain years. Version 3.0 of the US Forest Service (USFS) CONSUME fuel consumption model (Ottmar et al 2006, Prichard et al 2014) was used to calculate biomass fuel consumption, and emissions were estimated using data from the Fire Emission Production Simulator (FEPS) model (Ottmar et al 2007). All other emissions and inputs were consistent across both sets of simulations, including the use of the bi-directional NH_3_ flux scheme (Pleim et al 2013) with year-specific fertilizer application data. Further details about other aspects of the CMAQ model configuration are provided in Wilkins et al (2018).

We average the results from CMAQ over 5 years to capture both high and low fire years during that period, and to reduce the sensitivity of our results to other sources of year-to-year variability in wildland fire related deposition (e.g. precipitation, wind patterns, etc.). Since modeled wet deposition of N and S species simulated by the CMAQ has been evaluated against ambient data in previous assessments (e.g. Appel et al 2011, Zhang et al 2018), we do not include a detailed model evaluation in this study. Instead, we briefly evaluated model skill in accurately reproducing patterns of wet deposition across the U S during the study period (2008–2012). Our evaluation compared CMAQ results with observations from the National Atmospheric Deposition Program (NADP; nadp.slh.wisc.edu).

Figure S2 shows comparisons of annual wet deposition of nitrate (NO_3_^−^), ammonium (NH_4_^+^) and sulfate (SO_4_^2−^) simulated by CMAQ with observations from NADP. CMAQ estimated deposition correlates well (R > 0.8) for SO_4_^2−^ and NO_3_^−.^ CMAQ deposition of NH_4_^+^ underestimates the NADP observations by ~50% and shows weaker correlation (R = 0.63) during 2008–2012, which is consistent with conclusions from previous assessments (Zhang et al 2018) and is likely due to the well-documented challenges associated with representing NH3 emissions and cycling in atmospheric models (Bash et al 2013, Lee et al 2016). Dry deposition fluxes are more difficult to evaluate due to measurement scarcity (Williams et al 2017). Given the large contributions from dry deposition to total N fluxes from fires modeled in this work, future analyses would benefit from improved constraints on model representation of dry deposition processes.

It is important to note that modeled N deposition in CMAQ is highly sensitive to the treatment of bi-directional NH_3_ fluxes, which is an active area of model development (Bash et al 2013, Pleim et al 2019). Additionally, in this work we do not apply any bias correction to the modeled deposition fields (Zhang et al 2019); however, on average the CMAQ estimates of total deposition (TDEP) used in this work do not differ substantially from the fused model-measurement NADP TDEP (Schwede and Lear 2014) product over this same period. TDEP data and figures are accessible through the NADP website: http://nadp.slh.wisc.edu/committees/tdep/tdepmaps/.

### 2.2. Tree growth and survival data

We applied previously derived relationships between N deposition and tree survival, biomass, and size (Horn et al 2018) to estimate changes in above ground carbon due to estimated N deposition from wildland fire activity simulated by CMAQ. Horn et al (2018) presented quantitative relationships, derived from the U S Forest Service Inventory and Analysis Program (FIA; https://www.fia.fs.fed.us/) data, between total N deposition and both biomass growth and mortality rates at the species level. These relationships also include other important covariates: climate, tree size, and if identified as improving model comparison to observations, S deposition. The relationships with N deposition at the species level have a range of shapes (monotonic increasing, monotonic decreasing, hump shaped, and flat) and the slope of the relationship at a given level of N deposition can be used to calculate the sensitivity of growth or mortality for that level of N deposition. We focused on species identified in Horn et al (2018) as having N deposition variance inflation factors (VIF) of 10 or less indicating low collinearity with other environmental variables (16 species total in our study area). We also highlighted the species that were identified in Horn et al (2018) as having a more restrictive threshold for low collinearity (VIF < 3). Other studies have used similar relationships from Thomas et al (2010) to evaluate the sensitivity of forest demographics to N deposition in the Northeastern U S (Van Houtven et al 2019). We focused on areas where wildfire contributed 0.5 kg ha^−1^ yr^−1^ or more of either N or S, to examine areas where the impacts from fire are likely ecologically meaningful. Impacts may occur below 0.5 kg ha^−1^ yr^−1^, but given the uncertainty in several components of the analysis (e.g. the functional response curves in Horn et al (2018) for some species, the deposition values from CMAQ, etc.) we determined it was appropriately conservative to focus on areas with larger inputs from fire. Almost no areas received additional S deposition from fires above the 0.5 kg ha^−1^ yr^−1^ threshold, thus we focused on N for the rest of the analysis.

We estimated the effect on forest trees from wildfire primarily using the USFS FIA observed plot data. We first calculated the number and percent of trees of each species in the potentially affected areas (i.e. those experiencing N deposition from wildfire ≥ 0.5 kg ha^−1^). Then, using the functional response curves from Horn et al (2018), we calculated the growth and survival rate with and without wildfire. We then calculated the difference between these to represent the change in growth or survival associated with wildfire, holding all else equal.

## 3. Results

### 3.1. N and S deposition from wildland fires in the U S

**Figure 1.**
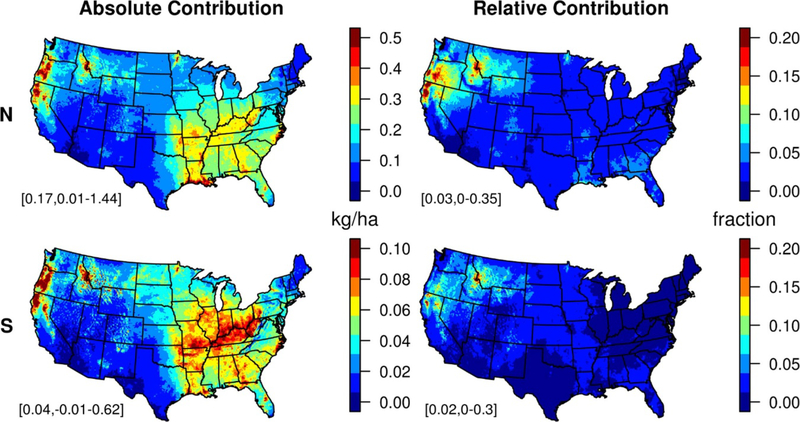
Absolute (left) and relative (right) contributions of wildland fires to CMAQ modeled deposition of N (top) and S (bottom) during 2008–2012. Domain means and ranges are shown in the bottom left of each panel.

We estimate that wildland fire contributed 0.2 kg N ha^−1^ yr^−1^ (5th–95th: 0.04–0.3) and 0.04 kg S ha^−1^ yr^−1^ (5^th^–95th: 0.006–0.08) across the contiguous US during 2008–2012 (figure 1). The largest effects from fire occurred in eastern Idaho, western Oregon, and northwestern California, where incremental N and S deposition exceeded 0.5 kg yr^−1^ and 0.1 kg yr^−1^ respectively. While parts of the eastern U S also experienced significant nutrient deposition from wildland fires, up to ~0.4 kg yr^−1^ N in some areas likely related to prescribed burning, the influence of fires on total N and S deposition compared to other sources is much lower in the east. Thus, the relative contribution of wildland fires to nutrient deposition is significantly higher in the Northwest compared to the rest of the country. Fire emissions account for ~10% of both total N and S deposition across much of the region, with maxima up to 30% in some areas. Spatial patterns vary for individual years (figures S3–S4).

**Figure 2.**
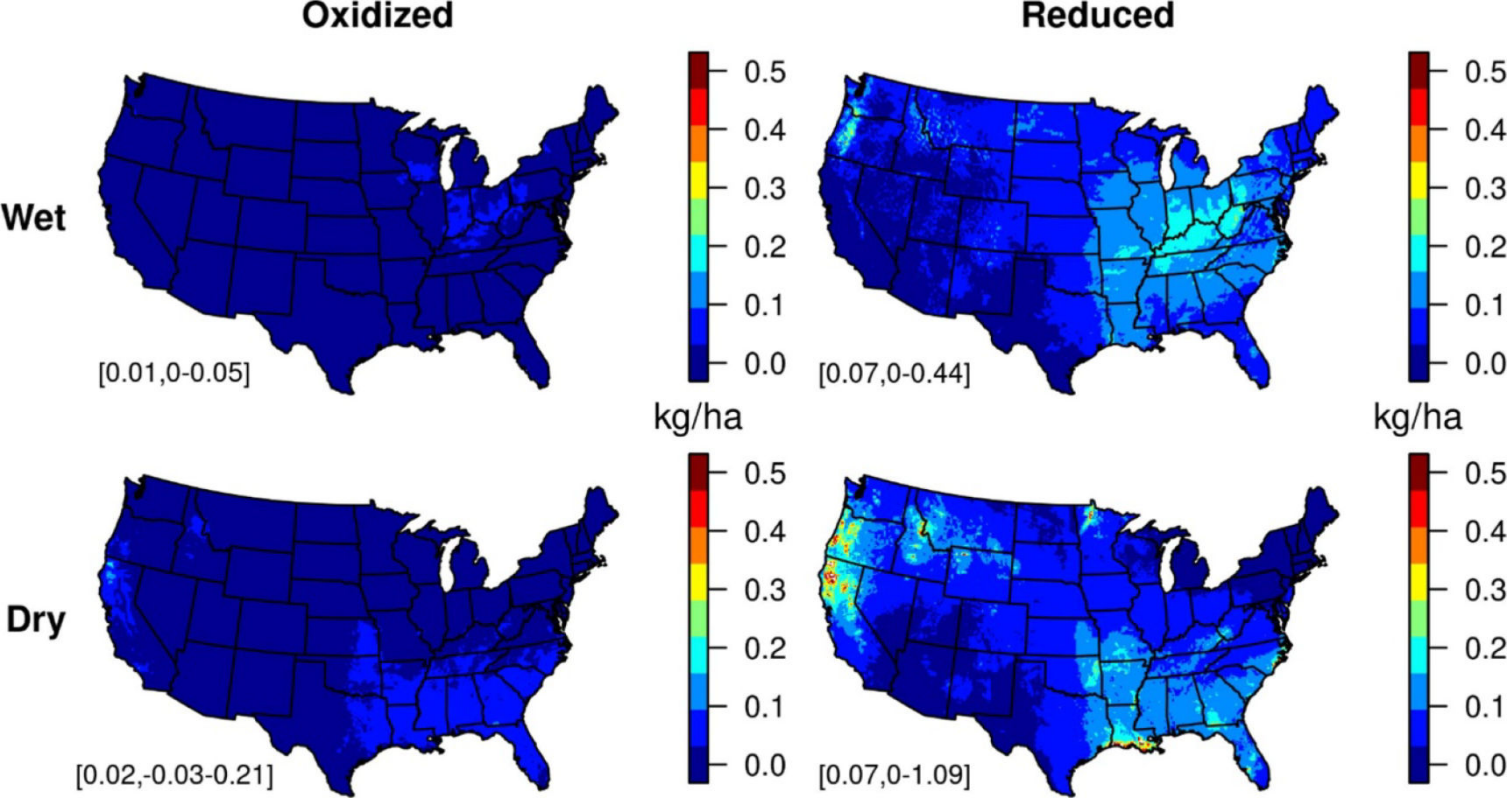
Contributions of wildland fires to CMAQ modeled wet (top) and dry (bottom) deposition of oxidized (left) and reduced (right) N during 2008–2012. Oxidized species include nitrogen oxides, nitrate, nitric acid, PAN, and organic nitrates; reduced species include ammonium and ammonia. Domain means and ranges are shown in the bottom left of each panel.

Modeled contributions to N deposition from fires are greatest from dry deposition of reduced N, contributing an average of 0.09 kg yr^−1^ (5^th^–95th: 0.03–0.23) across the Northwest (figure 2). While estimates of wet deposition compare well overall with observations during this time (figure S2), underestimated NH_4_^+^ deposition suggests that modeled deposition of both reduced and total N may be low in some areas. The relative contribution from reduced N has increased in the U S in recent decades and is expected to continue to increase in the future as NO_x_ emissions decline (Li et al 2016). In a future environment with lower levels of NO_x_ and potentially more frequent wildfires, our results suggest that this shift towards reduced N could be exacerbated further in high fire regions.

### 3.2. Impacts on tree growth and survival in the Northwest

We used the species functional response curves, i.e. the empirical relationships between N deposition and tree demographic rates, from Horn et al (2018) to quantify how wildland fire affected tree growth and mortality. We concentrated our ecological impact analysis on the Northwest region, where the relative contribution to estimated N and S deposition from wildland fires was highest. Within that study area there were 16 species that met the high confidence criteria for disentangling the N and S responses in the Horn et al (2018) analysis (i.e. VIF ≤ 10 for the correlation between N deposition and the other covariances that include climate and S deposition). There were 1075 FIA plots within the study area that experienced N deposition from fire exceeding 0.5 kg ha^−1^, containing 28 800 sampled FIA trees of these 16 species, representing^6^ 870 million total trees in the area. Growth and survival responses of these species to N deposition from wildland fires were estimated across the Northwest during 2008–2012 (table 1).

The gross effect was an increased growth rate for a population of ~623.7 million trees and decreased survival rate for a population of ~387.9 million trees (table 1). Douglas fir accounts for over 47% of trees in the study area (figure S5) and represents the largest number of affected trees in both cases (~415 million each), comprising over half of the positive growth response and the entirety of the negative survival response. Approximately 208 million other trees experiencing increased growth rates were spread across 11 species, including canyon live oak (~12.9 million trees) and western hemlock (~67.8 million trees). Five of these species also underwent a positive survival effect (totaling ~83.9 million trees), including Douglas fir (~27.7 million trees). Engelmann spruce was the only species to exhibit a positive effect on survival and a negative effect on growth, affecting the same ~2.1 million trees in both cases. Pacific silver fir was the only species to experience both a positive (~22.8 million trees) and a negative (~2.1 million) growth effect, without any response in survival.

**Figure 3.**
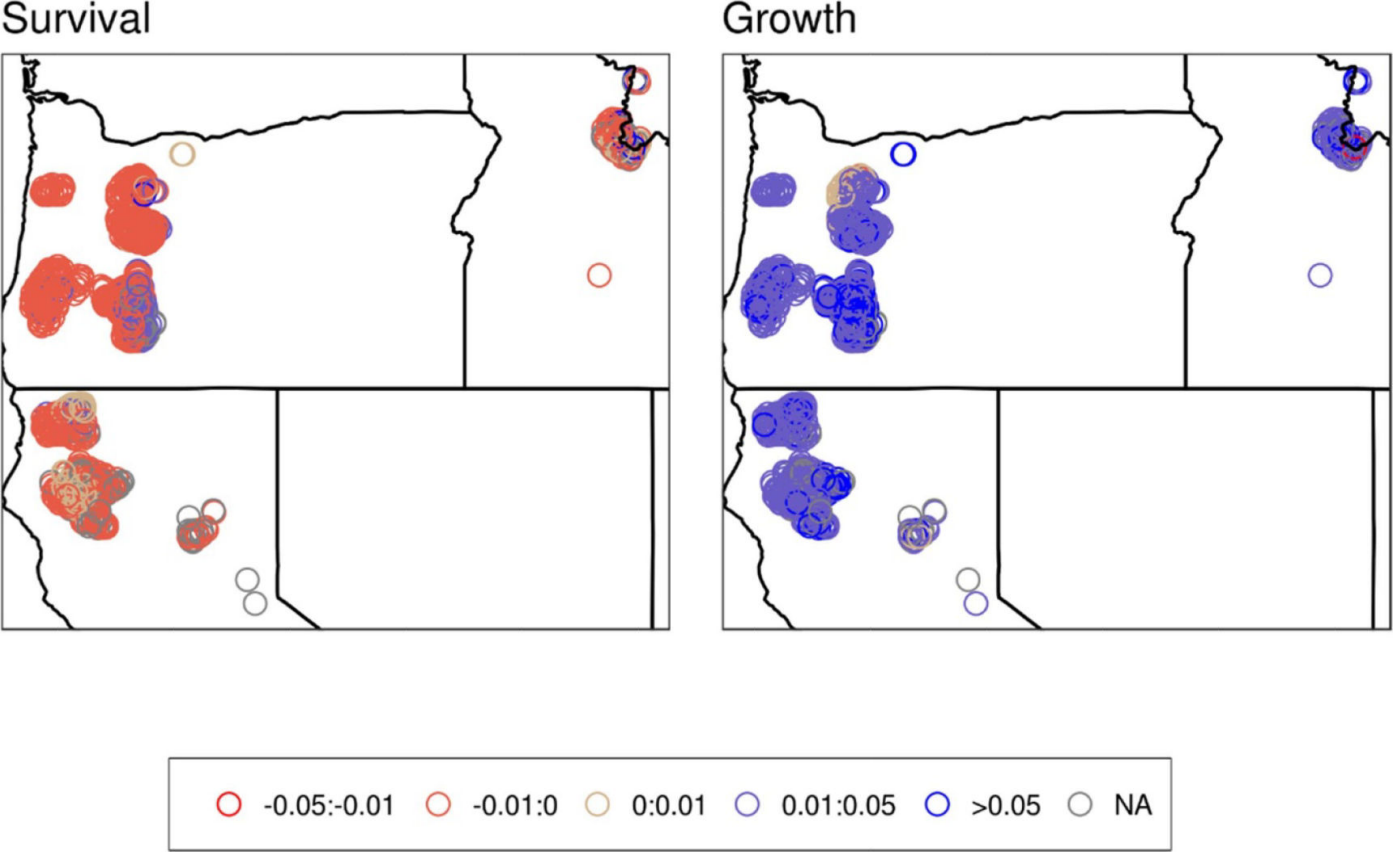
Median changes in the proportional rates of survival (left) and growth (right) in areas where modeled wildland fire N deposition exceeded 0.5 kg ha^−1^ yr^−1^. Each point represents one FIA plot. Values are unitless because they represent proportional changes.

**Figure 4.**
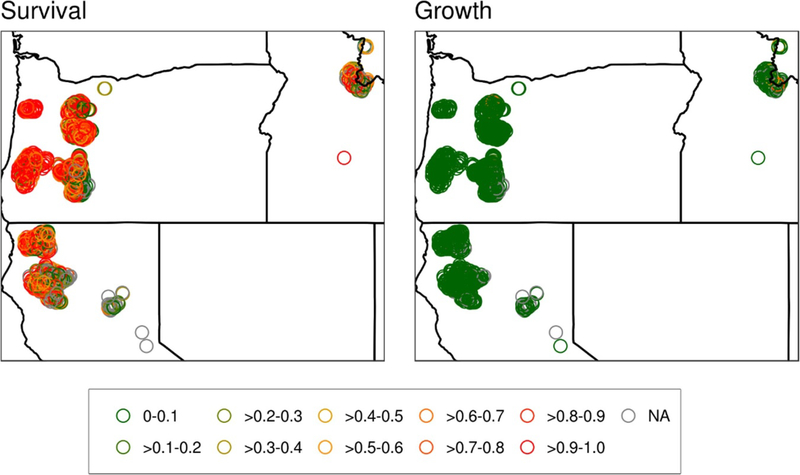
Same as figure 3 but for the fraction of trees in each FIA plot potentially impacted by wildland fire effects on survival (left) and growth (right).

Spatial patterns of both growth and survival of trees in the Northwest (figures 3–4) are largely driven by the response of the prevalent Douglas fir, which is widely distributed throughout the study region and constitutes the majority of both biomass and tree number in many of the FIA plots (figure S5). In Oregon, most of the areas had decreased median survival rates except for the eastern most edge of the sample area (figure 3). In contrast, in California and Idaho there was a mix of responses dominated by decreased survival. Across the study domain most sampled areas had increased median growth rates; however, there were a few areas with no effect and an area with decreased growth rates in Idaho. Similar spatial patterns were also observed for the proportional risk of basal area affected due to wildland fire with the exception that in the southernmost clusters of plots survival rates tended to increase. Some areas deviate from the broader regional patterns where Douglas fir is not as dominant, such as in the foothills/mountain valleys and higher elevations of the Cascade mountain range (Oregon) and Rocky Mountains (Idaho). The contrasting influence of the mountain hemlock leading to an overall positive tree survival response in central Oregon is particularly evident.

### 3.3. Overall effects of wildland fire across species

On average across all trees of these 16 species in the study area, the total effect of fire (table 2) increased survival by 0.01% and growth by 4.00%. Growth was increased 2.30%–38.24% across 13 species, decreasing growth only for the Engelmann spruce (−2.11%). Decreases in Engelmann spruce growth rates are potentially significant for high-altitude ecosystems, which are particularly sensitive to changes in climate and other environmental factors (Pepin et al 2015). The region-wide increase in survival was due to larger percent increases of less abundant species offsetting small percent decreases of the dominant species Douglas fir. Douglas fir is a very important tree species in this region, and thus small percentage decreases in survival over millions of individual trees can aggregate to regionally important effects. For example, the marginal effect of fire decreased survival on average for Douglas fir by ~0.37%, potentially putting at risk roughly 1.5 million trees. Recent stumpage price for Douglas fir in this USFS Region ($551/MBF in August 2019; https://www.dnr.wa.gov/programs-and-services/product-sales-and-leasing/timber-sales/timber-sale-querylog-prices) suggest a potential loss of ~$3.2 billion from wildfire (assuming ~3900 board feet per tree). Nonetheless, these predicted decreases in tree numbers were offset by larger percent increases (1.15%–17.66%) of less abundant species. Marginal effects of fire on growth rates were largest for tan oak (29.41%), grand fir (25.90%), and quaking aspen (38.24%), all species with slow overall growth rates, where even small changes in growth can significantly affect the total rate. These results agree with a recent study from California, which found a strong positive response of tan oak to N deposition (grand fir and quaking aspen were not analyzed in that study) (Fenn et al 2020). The responsiveness of growth rates to estimated fire effects for these slow- growing species highlights the sensitivity of such calculations to uncertainties in underlying assumptions related to estimating nutrient delivery from different sources (e.g. uncertainties in burned areas used in atmospheric models (Koplitz et al 2018)).

## 4. Discussion

While the immediate air quality impacts from wildland fires are visible and highly publicized, the broader environmental effects of wildland fire smoke are less obvious and understudied. Our results demonstrate that the ecological impacts from wildland fire smoke may be important in the Northwest, where wildland fires may have contributed as much as 30% of total N deposition during 2008–2012 accounting for ecologically significant amounts (>0.5 kg ha^−1^ yr^−1^) of N deposition across 4.2 million hectares. While notable, these estimated impacts reflect N contributions from fires averaged over 5 years; deposition and associated impacts during an individual high fire year can be over 20% larger (figure S2). Examining the more recent fire seasons of 2018 and 2020, which were some of the highest on record in the Northwest, would likely produce estimates of wildland fire N deposition much larger than those in this study.

We estimate that N deposition from wildland fire emissions during 2008–2012 potentially altered the growth and survival rates of over 27 000 trees measured by the FIA program, representing over 870 million total trees in the Northwest region. While the average response was a positive fertilization effect, there was a range in estimated responses, with some species experiencing negative survival and growth effects. Regardless, positive and negative effects co-occurring suggest that wildfire may contribute to compositional changes across the forest landscape, which could affect various ecological relationships and ecosystem services derived. Given the large amount of fire activity in the Northwest, both increased tree growth and reduced tree survival could exacerbate future fire risk in these already fire prone areas, although relationships between fuel loading and fire activity are complicated and difficult to predict. Additionally, the relatively high frequency variability in nutrient loadings from wildland fires may present conditions not fully captured in existing critical load relationships developed from decadal scale observations. The response of ecosystems to rapid loadings from fires warrants further investigation.

In addition to N and S, fires are also a source of atmospheric phosphorous (P) (Zhang et al 2002, Mahowald et al 2005, Sundarambal et al 2010, Vicars et al 2010, Barkley et al 2019). Recent work suggests that P concentrations are increasing at remote monitoring sites across the country, and that atmospheric sources may contribute to this trend (Stoddard et al 2016). Several studies have used model-based approaches to estimate atmospheric P concentrations globally (Mahowald et al 2008, Wang et al 2015). However, rigorous treatment of P-containing particles is missing from most atmospheric models (including CMAQ) due to limited observational constraints, which is why we did not estimate the contribution of wildland fires to atmospheric P deposition in this analysis. Given the ecological importance of understanding and monitoring atmospheric P fluxes, the representation of P-containing compounds from all sources in atmospheric models warrants further attention.

We also acknowledge that our analysis is limited in several notable ways. First, we examine only 5 years of fire-related impacts (2008–2012). Examination of a longer period could provide a more robust understanding of the relationships between wildland fires, nutrient deposition, and ecosystem impacts discussed in this work. Next, while variability in fuel loading characteristics is considered in the CONSUME model, the underlying land use classifications are static in both the WRF meteorological model and the CMAQ simulation. Consequently, there was no online adjustment for wildfire impacts on evapotran- spiration or leaf area index, properties that would affect both biogenic emissions and deposition in the model. A longer-term study using a dynamic vegetation model to investigate coupled land surface-air quality interactions related to wildland fire activity would be informative for future work. Additionally, many of the largest impacts quantified in this analysis occur in complex terrain, which is challenging for air quality models to represent. Most existing air pollution monitoring sites are located in populated areas with elevated air pollutant concentrations. While the ongoing development of the CMAQ model benefits from extensive evaluation across a broad user community for a range of applications, a more comprehensive evaluation of CMAQ performance in the context of N deposition in sparsely populated, fire prone areas would be of interest to inform similar analyses in the future. Applying the standard FIA expansion factors to estimate total trees affected may also contribute some uncertainty to our results. The FIA expansion factors estimate the number of trees at the county level that a measured tree may represent. Since deposition across the county may differ from that at the plot, especially for western counties that are large, this expansion may introduce uncertainty. However, since deposition across the county may be expected to be higher than at the plot as much as it may be lower (i.e. there is no expectation of bias), we do not expect this to introduce significant bias in the results, though specific spatial patterns may be affected. Finally, this analysis does not quantify the full range or magnitude of longer-term wildfire impacts on ecosystems, but rather assesses whether wildland fires comprise a significant source of nutrients relative to anthropogenic sources in some areas. A multi-decadal assessment would be more robust for comprehensively characterizing the influence of wildland fire deposition on ecosystems, as would a more thorough investigation of the relative impacts of naturally occurring wildfire compared to the influence of specific anthropogenic processes on nutrient deposition and ecosystem dynamics. Both types of assessments would benefit prospective analyses of how the role of wildland fire might change in the future, e.g. projections of expected landscape response due to future changes in climate.

Minimizing the air quality effects of wildland fires is a primary consideration in current fire management practices (National Association of State Foresters and Coalition of Prescribed Fire Councils 2018); however, accounting for and effectively controlling the overall environmental impact of fires is more challenging. As mentioned above, fires play an important role in many U S ecosystems (Hutto et al 2016), and prescribed burns are a critically important tool for mitigating the occurrence of catastrophic wildfire events, particularly as the WUI expands and becomes more populated (Radeloff et al 2018). However, excess nutrient contributions from anthropogenic sources (mostly fossil fuel combustion and industrial agriculture) have created situations where many ecosystems may already be N-saturated (Aber et al 1998), and additional atmospheric deposition from fires can lead to nutrient imbalances that may be harmful for ecosystems, as our results suggest. If wildland fire risk increases in parts of the West as projected (Ford et al 2018), the use of controlled burns as a wildfire abatement mechanism could also become more frequent. Reconciling the tradeoffs of prescribed fire as a land management tool, some of which are highlighted in this work, presents just one of many ongoing challenges for land managers in the rapidly evolving landscape of the West. More work to comprehensively assess and anticipate the full range of interactions between wildland fires and the broader earth system is needed to help develop management strategies that effectively minimize fire damage while also protecting public health and natural ecosystems.

## Supplementary Material

Supplement1

## Figures and Tables

**Table 1.** Estimated impact of N deposition from wildland fire on 16 tree species, ordered from highest to lowest number of sampled trees. Shown are the number of sampled trees in FIA plots, and in parentheses the number of trees (in millions) that those represent across the landscape. Tree survival and growth either decreased (−), increased (+), or was unaffected (0).

Species			Survival			Growth		
Common name	Latin name	N trees (mil.)	−	+	0	−	+	0

Douglas-fir^a^	*Pseudotsuga menziesii*	14 383 (415.6)	13 504 (387.9)	879 (27.7)			14 383 (415.6)	
Western hemlock	*Tsuga heterophylla*	2767 (67.8)			2767 (67.8)		2767 (67.8)	
White fir^a,b^	*Abies concolor*	2293 (60.4)			2293 (60.4)			2293 (60.4)
Lodgepole pine^a^	*Pinus contorta*	1762 (65.1)			1762 (65.1)			1762 (65.1)
Mountain hemlock	*Tsuga mertensiana*	1648 (39.1)		1648 (39.1)			1648 (39.1)	
Canyon live oak	*Quercus chrysolepis*	1563 (86.5)			1563 (86.5)		259, *1304*^c^ (12.9, *73.6*)	
Pacific silver fir^a^	*Abies amabilis*	1056 (24.9)			1056 (24.9)	72 (2.1)	984 (22.8)	
Ponderosa pine ^a,b^	*Pinus ponderosa*	879 (29.0)			879 (29.0)			879 (29.0)
Incense-cedar ^a,b^	*Calocedrus decurrens*	735 (17.8)			735 (17.8)		700, *35*^c^ (16.4, *1.4*)	
Western redcedar	*Thuja plicata*	587 (10.0)			587 (10.0)		587 (10.0)	
Subalpine fir^a^	*Abies lasiocarpa*	367 (21.6)			367 (21.6)		367 (21.6)	
Tanoak	*Lithocarpus densiflorus*	352 (15.5)			352 (15.5)		36, *316*^c^ (2.3, *13.2*)	
Grand fir^a,b^	*Abies grandis*	332 (14.1)		332 (14.1)			332 (14.1)	
Engelmann spruce^a,b^	*Picea engelmannii*	64 (2.1)		64 (2.1)		64 (2.1)		
Quaking aspen ^a^	*Populus tremuloides*	9 (0.9)		9 (0.9)			9 (0.9)	
Western larch ^a,b^	*Larix occientalis*	5 (0.1)			5 (0.1)		5 (0.1)	
All species		28 802 (870.5)	13 504 (387.9)	2932 (83.9)	12 366 (398.7)	136 (4.2)	22 077 (623.6)	4934 (154.5)

a Indicates that the N–S correlation was relatively high (≥0.6).

b Indicates that the VIF was relatively high (≥3.0).

c Numbers in italics correspond to trees for which once N deposition from fire was removed, the deposition was below the minimum experienced across the range in Horn *et al* (2018). There were three species for which this occurred. We assume that the basic relationship (increase/decrease/none) holds below the minimum since the effect from fire was quantitatively small (figure 1, 99th percentile was 1.07 kg, thus almost 99% of trees experienced a shift of <1 kg).

**Table 2.** Change in survival and growth rates due to N deposition from fire averaged for all 16 species included in the study.

Common name	Mean % change in survival due to fire (min, max)	Mean % change in growth due to fire (min, max)

Douglas-fir	−0.37 (−1.03, 0.34)	2.3 (0.75, 4.86)
Western hemlock	0	6.83 (0.50, 17.58)
White fir	0	0
Lodgepole pine	0	0
Mountain hemlock	2.45 (0.52, 4.72)	5.06 (1.86, 8.70)
Canyon live oak	0	13.02 (4.99, 18.97)
Pacific silver fir	0	6.48 (−1.07, 15.50)
Ponderosa pine	0	0
Incense-cedar	0	5.07 (1.35, 8.66)
Western redcedar	0	4.31 (2.11, 8.02)
Subalpine fir	0	5.66 (3.33, 7.32)
Tanoak	0	29.41 (23.66, 33.57)
Grand fir	1.15 (0.43, 1.77)	25.9 (15.02, 44.00)
Engelmann spruce	17.66 (8.02, 22.17)	−2.11 (−2.54, −1.24)
Quaking aspen	2.75 (2.75, 2.75)	38.24 (38.24, 38.24)
Western larch	0	4.67 (3.95, 7.57)
**Tree average**	**0.01**	**4.00**

## Footnotes

6 Each tree in the FIA is representative of a larger set of U S forest trees.
