# Supplementary material for: The contribution of wildland fire emissions to deposition in the U S: implications for tree growth and survival in the Northwest
Source: Environ Res Lett. Author manuscript; Available in PMC 2022 Jan 29. (PMC7970516; doi:10.1088/1748-9326/abd26e)
Supplement: Supplement1 [file NIHMS1669185-supplement-Supplement1.pdf]

Supporting Information for:

The contribution of wildland fire emissions to deposition in the U.S.: Implications for tree growth and survival in the Northwest

Shannon N. Koplitz[1,†*], Christopher G. Nolte[1], Robert D. Sabo[2], Christopher M. Clark[2], Kevin J. Horn[3], R. Quinn Thomas[3], and Tamara A. Newcomer-Johnson[4]

[1] Center for Environmental Measurement and Modeling, US EPA, Research Triangle Park, NC
[2] Center for Public Health and Environmental Assessment, US EPA, Washington, DC
[3] Department of Forest Resources and Environmental Conservation, Virginia Tech, Blacksburg, VA
[4] Center for Environmental Measurement and Modeling, US EPA, Cincinnati, OH

[*] Corresponding author: Koplitz.shannon@epa.gov
[†] Current address: Office of Air Quality Planning and Standards, US EPA, Research Triangle Park, NC

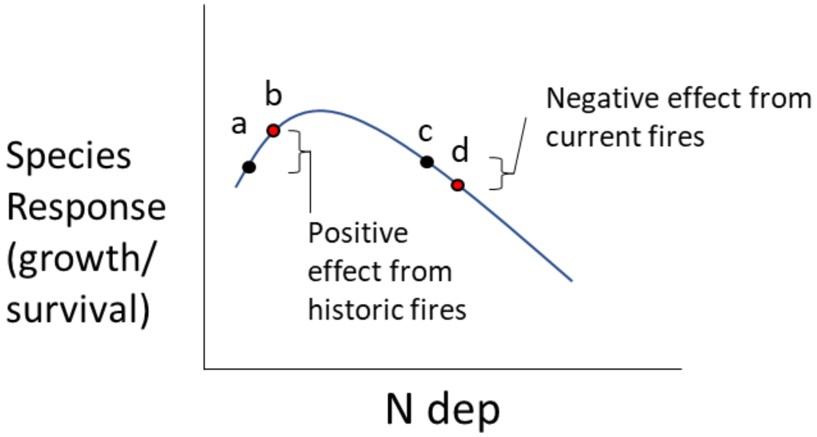

Figure S1. Working hypothesis: N deposition historically from fires was low, and so the incremental effect from fire was generally positive (i.e. moving from point a to b). Contemporary N deposition is much higher, thus the incremental effect from fire is now negative (i.e. moving from point c to d).

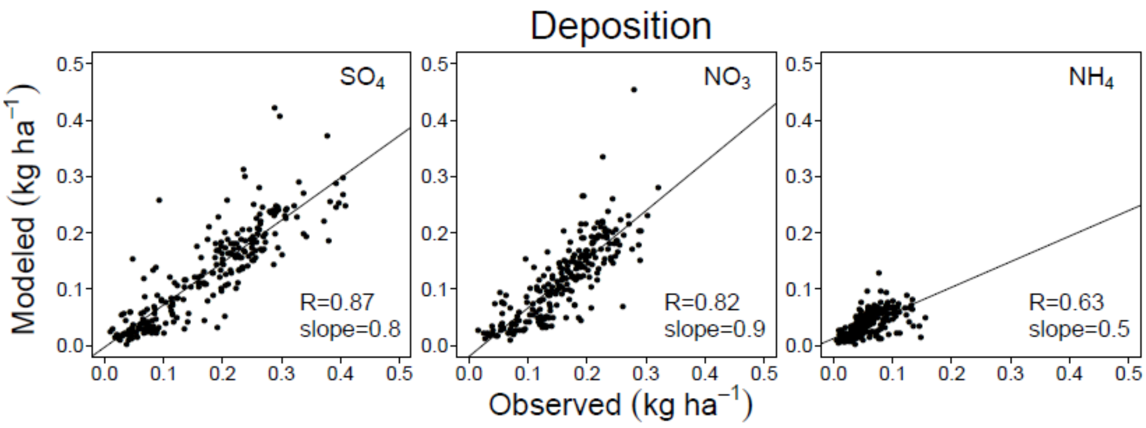

Figure S2. Wet deposition fluxes modeled with CMAQ compared to observed values from NADP during 2008-2012. Values represent 5-year averages at each NADP monitoring site location. Correlation coefficients and slopes for each comparison are shown inset.

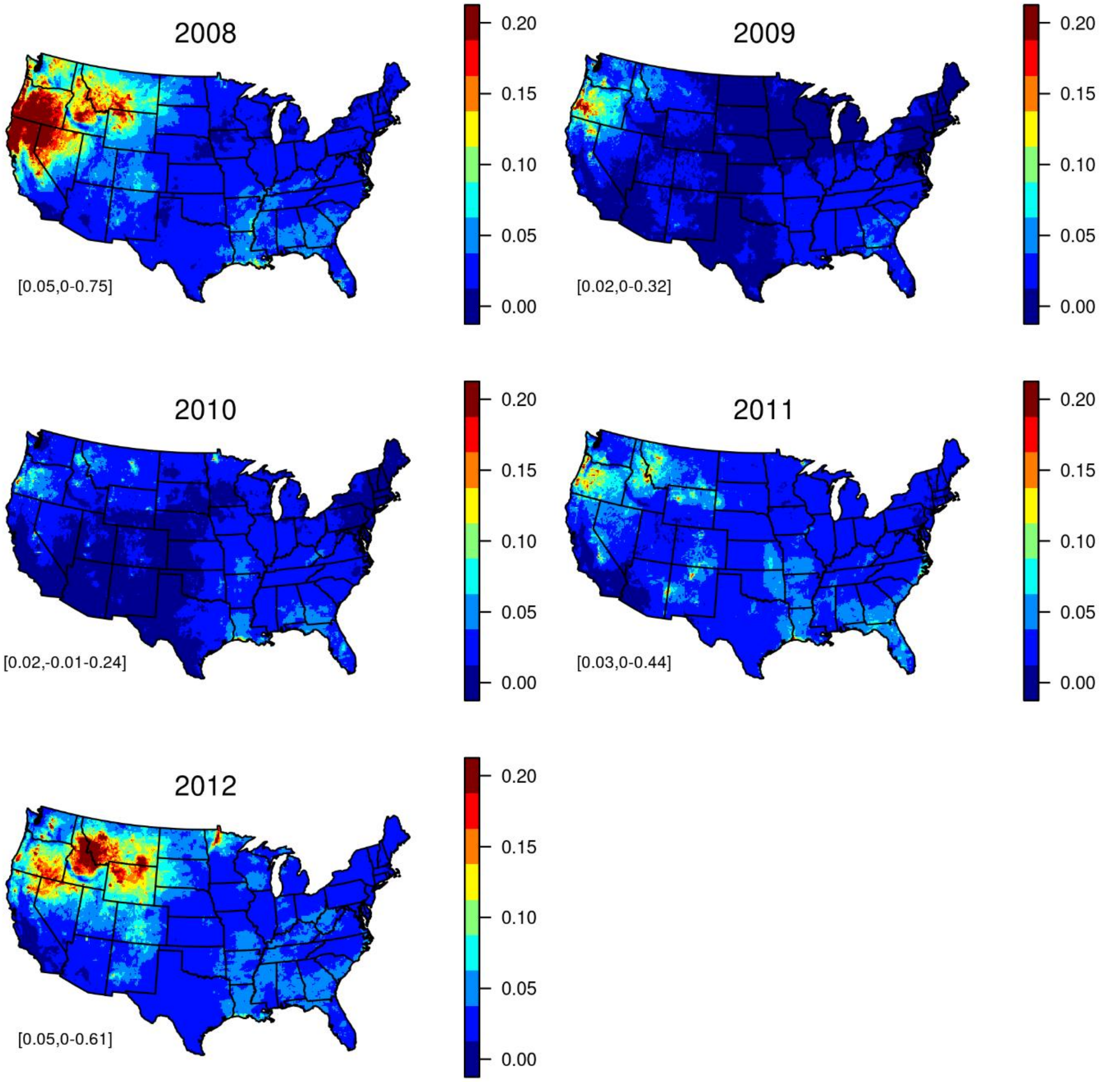

Figure S3. Fraction of total N deposition due to wildland fires during 2008-2012 simulated with CMAQ. Domain wide means and ranges are shown inset in brackets.

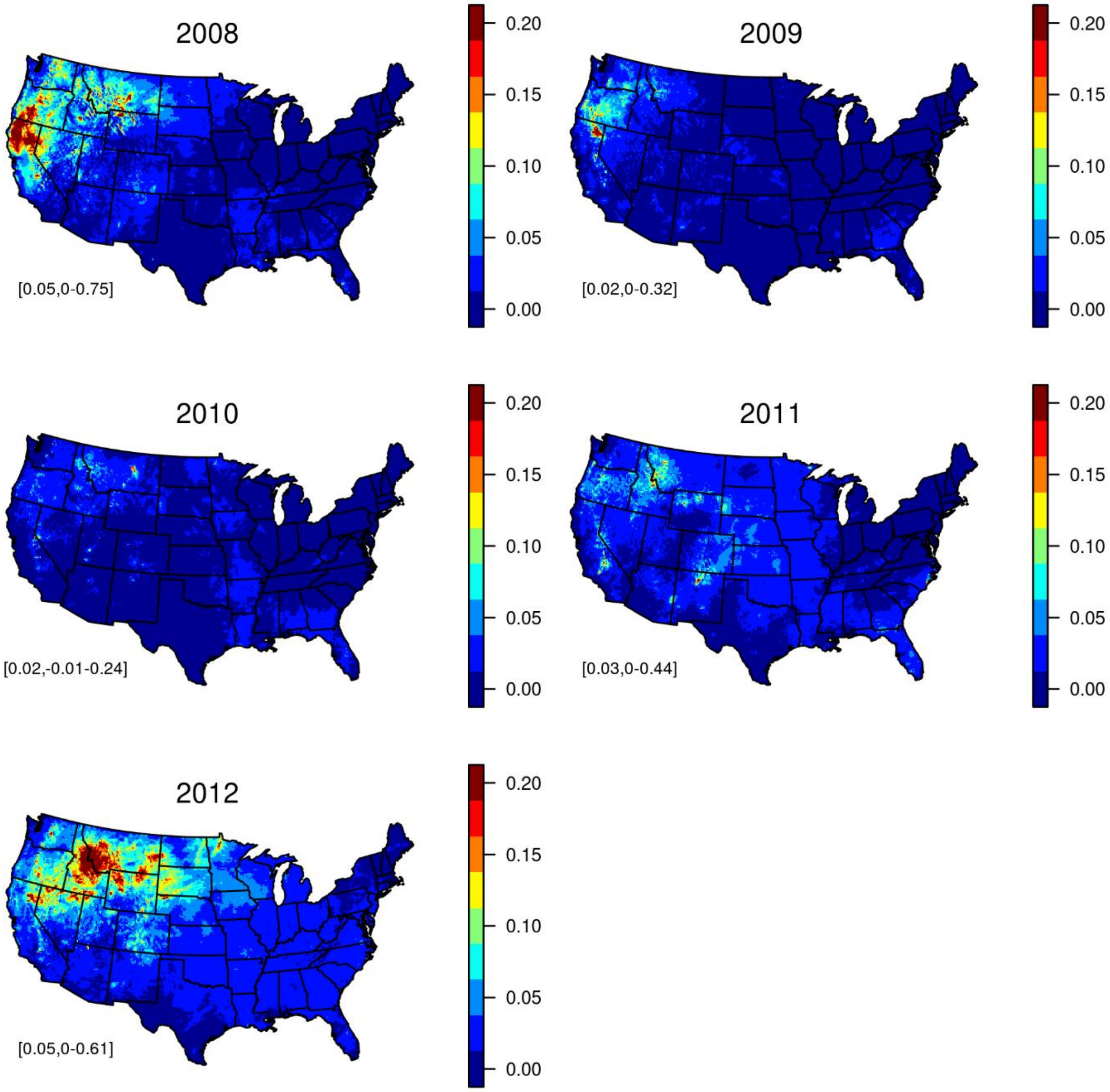

Figure S4. Fraction of total S deposition due to wildland fires during 2008-2012 simulated with CMAQ. Domain wide means and ranges are shown inset in brackets.

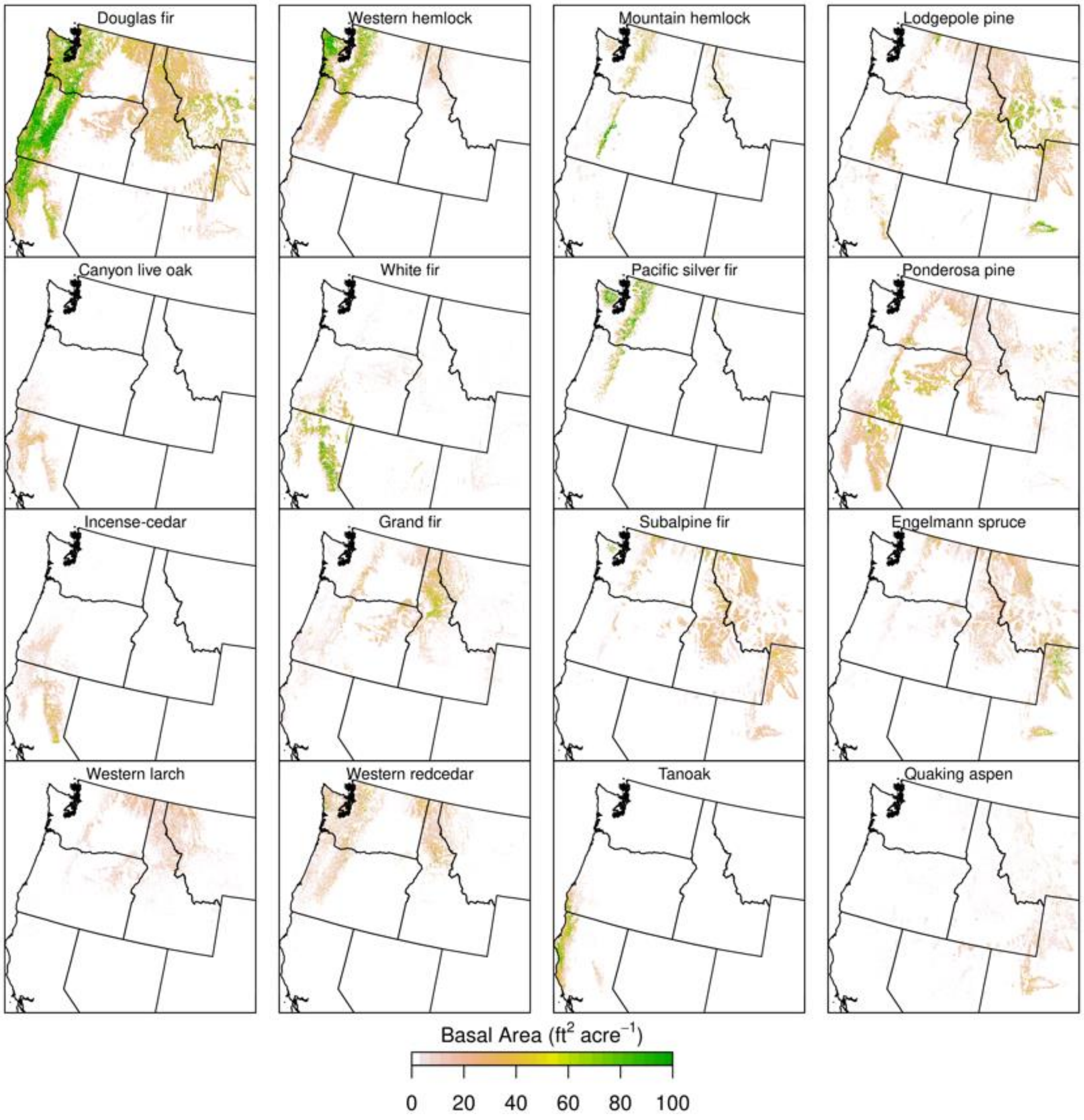

Figure S5. Basal area density distribution for species included in this work. Distributions shown here are from Wilson et al. (2013).

Wilson, B. T., Lister, A. J., Riemann, R. I., & Griffith, D. M. (2013). Live tree species basal area 641 of the contiguous United States (2000-2009).
